# Development of Cutaneous Leishmaniasis after Leishmania Skin Test

**DOI:** 10.1155/2011/631079

**Published:** 2011-11-24

**Authors:** Paulo R. Machado, Augusto M. Carvalho, Gustavo U. Machado, Marina L. Dantas, Sérgio Arruda

**Affiliations:** ^1^Serviço de Imunologia, Hospital Universitário Prof. Edgard Santos, Universidade Federal da Bahia, Salvador, BA 40000, Brazil; ^2^Instituto Nacional de Ciência e Tecnologia de Doenças Tropicais, CNPq/MCT, Salvador, BA 40000, Brazil; ^3^Cursos de Medicina e Biomedicina, Escola Bahiana de Medicina e Saúde Pública, Salvador, BA 40000, Brazil; ^4^Centro de Pesquisas Gonçalo Moniz, LASP, Fundação Oswaldo Cruz, Salvador, BA 40000, Brazil

## Abstract

Thirty-year-old female with a previous history of a cutaneous ulcer suspicious of leishmaniasis 20 years ago presented with a new complaint of a depressed papular lesion 8 × 7 mm in the right lower extremity. The lesion was of 10-day duration. Because early cutaneous leishmaniasis (CL) lesions may have a non-ulcerated appearance, a Leishmania skin test (LST) was performed on the forearm with a strong positive result (38 × 32 mm). After 8 days, the lesion in the leg, which was diagnosed as folliculitis, completely healed. However, a typical CL ulcer (26 × 24 mm) developed at the LST site. Histopathology of the new lesion did not identifiy parasites, but the findings were consistent with a diagnosis of CL. Further analysis identified amastigotes by immunohistochemical stain. Mononuclear cells harvested from the patient were stimulated with Leishmania antigen and showed high levels of production of both tumor necrosis factor-alpha (TNF-**α**) and interferon-gamma (IFN-**γ**): 2,943 pg/mL and 2,313 pg/mL, respectively. After 40 days of treatment with antimony and pentoxifylline, the ulcer resolved. The development of CL at the LST site suggests a strong Th1 immune response, and it is an *in vivo* documentation of the role of the host immune response in the pathology of CL. It teaches us that LST should be cautiously, if at all, used in patients with self-healing CL ulcers.

## 1. Introduction

Cutaneous leishmaniasis (CL) due to *Leishmania braziliensis* is characterized by a well-delimited ulcer with raised borders and localized mainly on the inferior limbs [1, 2]. In CL, parasites are not easily found in the lesion. The sensitivity of serological tests is low. However, the predictive value of the delayed-type hypersensitivity Leishmania skin test (LST) is high [3, 4]. Thus, when parasites are not detected, a positive LST accompanied by typical ulcer and histopathological findings compatible with leishmaniasis is accepted as diagnostic standard for CL in endemic areas [3]. The test is considered safe, and its side effects are mild [4]. Rarely topical steroids are needed to attenuate pain, erythema, or eruptive lesions that appear after the test is administered. Both parasite and host immunological factors participate in the pathogenesis of CL. Although the ability to mount a type 1 immune response to Leishmania is associated with protection [5], in *L. braziliensis* infection, the tissue damage is associated with an exaggerated cell-mediated immune response [6, 7]. This case represents *in vivo* evidence of the host immune response in the pathogenesis of CL ulcers.

## 2. Case Presentation

**Figure 1:**
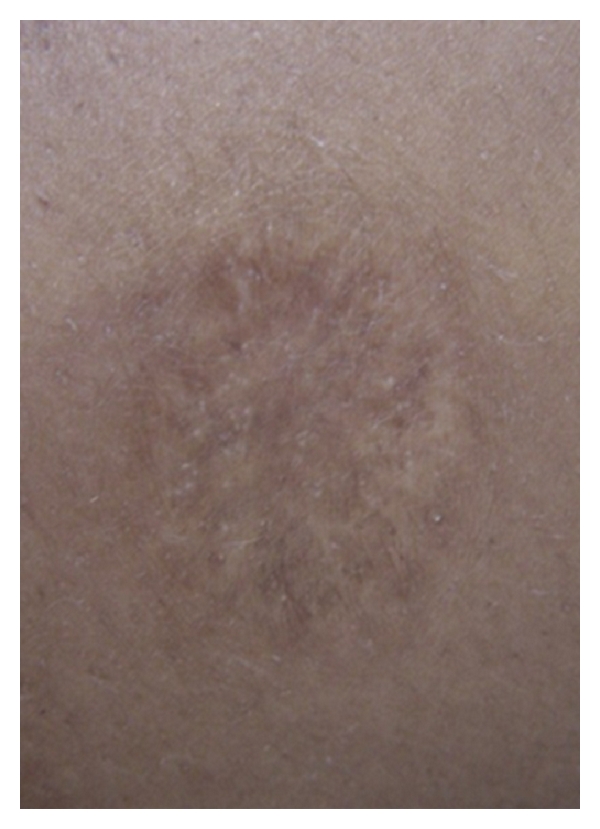
A well-delimited atrophic scar in the left thigh measuring 30 × 20 mm.

**Figure 2:**
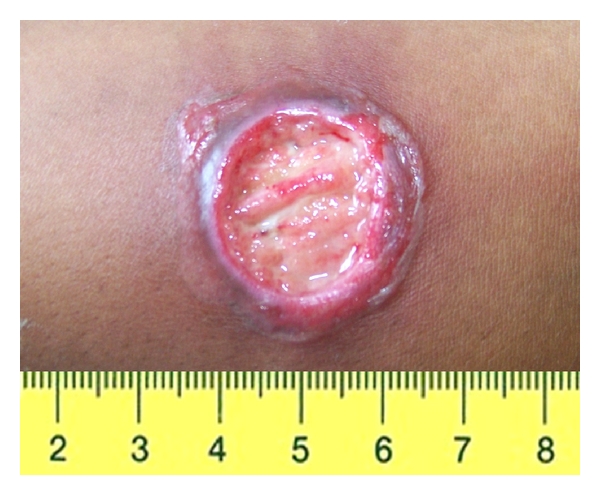
Cutaneous round ulcer with raised borders measuring 26 × 24 mm in the forearm where the Montenegro skin test was performed.

**Figure 3:**
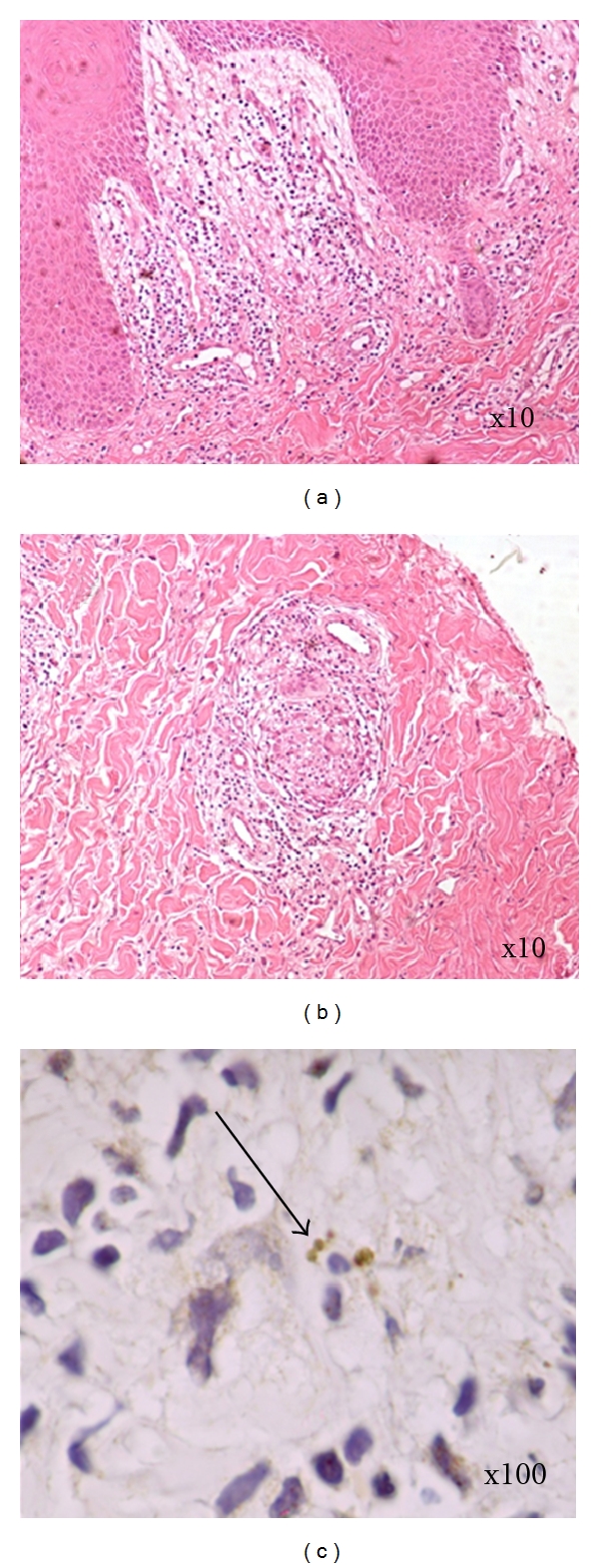
Hematoxylin-eosin staining of biopsy. (a) Edema and chronic inflammation in the papillary dermis (10x). (b) Granuloma in the dermis (10x). (c) Immunohistochemistry staining of *L. braziliensis* amastigotes ((100x) arrow).

RJS, a 30-year-old woman, was seen at the health post of Corte de Pedra, Tancredo Neves—Bahia, a reference center for diagnosis and treatment of CL. The patient complained of a small papular lesion in her right leg developing over seven to 10 days. When she was 10 years of age, she had an ulcer in the left thigh suspicious of CL that spontaneously healed in about 4 months. Given her prior history of ulcer, she decided to seek help at the health post when the new lesion appeared. On physical examination, a depressed papular lesion measuring 8 × 7 mm in the right leg suspicious of folliculitis was noted. There was also a well-defined atrophic scar in the left thigh, measuring 30 × 20 mm (Figure 1). There was no lymphadenopathy. Because early CL lesions in *L. braziliensis* infection may have a papular and nonulcerated aspect [8] and the patient lived in an endemic area, the diagnosis of CL was suspected. LST was prepared as previously described [9]. Briefly promastigotes of *L. amazonensis* were disrupted initially by multiple freeze and thaw and later by sonication. The material was adjusted to 250 *μ*g/mL with sterile PBS containing Tween 80 and phenol [9]. An intradermal injection of 0.1 mL was administered in her left forearm. After 48 hours, the intradermal reaction was highly positive with an induration of 38 × 32 mm. Eight days after the skin test, while the folliculitis in the right leg disappeared, a cutaneous round ulcer with raised borders measuring 26 × 24 mm, typical of leishmaniasis, was observed in the site where the LST was administered (Figure 2). The clinical picture was typical of an ulcerated CL lesion, but the culture of the aspirated material from this lesion was negative for Leishmania. The histopathological analysis (Figure 3) showed chronic perivascular inflammation with granuloma formation and the presence of neutrophils, lymphocyte, monocyte, and plasma cells. Parasites were not identified, but a few amastigote forms were detected by immunohistochemical analysis performed with rabbit anti-*Leishmania braziliensis *antibody in skin sections. 

**Figure 4:**
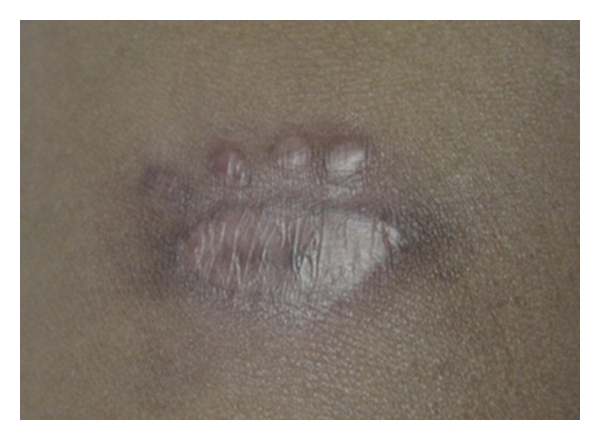
Scar in the forearm 40 days after the initiation of therapy.

Determination of cytokine production was performed by ELISA (R&D Systems, Minneapolis, Minn, USA) in supernatants of peripheral blood mononuclear cells stimulated with soluble Leishmania antigen (SLA) for 72 hours [6]. TNF-*α* and IFN-*γ* levels were 47 pg/mL and 0 pg/mL, respectively, in unstimulated cultures and were 2,943 pg/mL and 2,313 pg/mL, respectively, in SLA-stimulated cultures. These values were similar to those detected in 3 patients with CL who were evaluated on the same day: TNF-*α*: 2,139 ± 953.8; IFN-*γ*: 3,653 ± 312.6. The patient was treated with pentavalent antimony (Glucantime) in a dosage of 20 mg/kg/day and pentoxifylline 400 mg tid for 20 days. Pentoxifylline has anti-inflammatory properties, and it has been previously shown that its use in combination with antimony is more effective than antimony alone in the treatment of tegumentary leishmaniasis [10, 11]. The ulcerated lesion in the left forearm was completely healed 40 days after the initiation of therapy (Figure 4).

## 3. Discussion

This paper calls attention to a patient who had a past (20 years prior) self-healing ulcerated lesion compatible with CL and who developed a typical ulcer of CL in the place where a LST was performed. Although parasites were found in the immunohistochemical analysis, there are strong indications that the cutaneous ulcer observed in this patient was due predominantly to the skin test with Leishmania antigen: (1) despite that parasites DNA are found in the scar of patients cured of tegumentary leishmaniasis, reactivation is uncommon except in patients with HIV [12]; (2) the development of cutaneous ulcer caused by *L. braziliensis* usually takes 1-2 months and it is preceded by lymphadenopathy, papular or nodular lesion, and a superficial ulcer, features not observed in this patient [8, 13]; (3) the lesion occurred in the same place where the skin test was performed; (4) while classical CL ulcers heal after 60 or 90 days of antimony therapy [13], the cutaneous ulcer presented here healed in less than 40 days. Together, these data suggest that CL developed in this patient was due to a skin test performed in the location.

Host and parasite factors participate in the pathogenesis of tegumentary leishmaniasis [2, 6, 7]. The persistence of the parasites in patients with self-healing CL is important to maintain the T cell response after clinical cure. It is interesting that, in this case, amastigotes were found in a place where the LST was performed. Prior studies have shown parasite DNA in scars of CL patients [14], but herein we found amastigotes in the tissue where the new ulcer developed. These parasite forms were not present in the LST because it was prepared with promastigotes, and as previously described it contains only SLA [9]. Moreover, there is strong evidence for the role of T cell responses in the pathogenesis of *L. braziliensis* infection including (1) a strong inflammatory reaction in tissue in the absence or with very few parasites in the lesion [15]; (2) a correlation between frequency of CD4^+^ T cells expressing IFN-*γ* and TNF-*α* and size of the lesion [7]; (3) a correlation between CD4^+^ T cell activation markers expression and size of the lesion [7]; (4) presence of cytotoxicity in preulcerative lesions [16]; (5) a correlation between granzyme expression by CD8 T cells and the inflammatory reaction in the tissue [17]; (6) development of ulcers despite antimony therapy in patients diagnosed with early and preulcerative CL [8, 13]. 

In the present case, the patient had a history of a previous ulcer that self-healed. Self-healing of CL usually occurs after 6 months of the initiation of the ulcer [18]. Immunological studies in patients with self-healing CL showed that there are a high lymphocyte proliferation and high production of IFN-*γ* in cultures stimulated with SLA [18]. The presence of a strong type 1 immune response in subjects with self-healing disease is probably due to the presence of the parasite and the persistent stimulation of the immune response. The immune response in the young lady presented in this paper was similar to that observed in patients with active CL, despite the fact that her leishmaniasis lesion had occurred 20 years previously. In such case, the development of a typical CL ulcer after the intradermal injection of Leishmania antigen could be explained by the activation of antigen-reactive T cells that, after challenge, induced tissue damage at the site the antigen was inoculated. The patient was treated with antimony plus pentoxifylline, a TNF-*α* inhibitor in part because of the previous observation that combination therapy was more effective [10, 11] and also because we believe that the inflammatory response played an important role in the development of the ulcer at the site where LST was applied. It is known that TNF-*α* plays a key role in controlling parasite multiplication in the initial phase of Leishmania infection [19], but it is also associated with ulcer development [6, 7]. Therefore, it is possible that modulation of the immune response by pentoxifylline improved the therapeutic response in this patient [10, 11].

This paper emphasizes the role of the immune response as a cause of the tissue damage in CL. Moreover it illustrates important clinical lessons: first, that antimony therapy should be provided even for patients who present with self-healing CL secondary to *L. braziliensis* infection; second, that the LST should be used with caution, if at all, in patients with self-healing CL.
